# Enhancing Early Detection of Mild Cognitive Impairment Using Preprocessed Connected Speech and Natural Language Processing

**DOI:** 10.1002/alz.095323

**Published:** 2025-01-09

**Authors:** Chingching Lu, Pei‐Ning Wang

**Affiliations:** ^1^ National Tsing Hua University, Hsinchu City, Taiwan Taiwan; ^2^ Taipei Veterans General Hospital, Taipei Taiwan

## Abstract

**Background:**

Early detection is crucial for the timely intervention and management of dementia, potentially slowing its progression. Early stages of dementia might only subtly affect communication, yet connected speech analysis can detect these minor anomalies. Cognitive tests involving connected speech, like the Boston Diagnostic Aphasia Examination’s (BDAE) “cookie‐theft” picture description task, are pivotal in detecting dementia. However, previous research typically treats their responses as a whole.

**Method:**

This study preprocesses connected speech from the “cookie theft” task to facilitate AI‐driven detection improvements. We analyzed narratives from 54 normal subjects, 26 with MCI, and 38 with Alzheimer’s Disease (AD), focusing on nine major concepts to quantify accuracy across groups.

**Result:**

Differences in concept accuracy were significant even among normal individuals. A detailed analysis revealed that concepts 3 and 1 were particularly effective in differentiating between normal individuals and those with cognitive impairments, and between MCI and AD. Concepts 2, 3, and 7 were crucial for distinguishing between normal and MCI subjects. Concepts 4, 5, 8, and 9 showed no discriminatory power.

**Conclusion:**

Advances in technology, particularly in natural language processing (NLP), allow for the automated analysis of connected speech. It can provide quick and reliable assessments. By categorizing and preprocessing speech data before AI analysis, our approach enhances the model’s predictive accuracy for early‐stage dementia detection. It can also help for developing more diagnostic images and cognitive training exercises. Furthermore, it does not require expensive equipment or invasive procedures, making it accessible for widespread use in various settings, including community centers and primary care, and particularly beneficial for regular monitoring.

